# A Conceptual Framework of Person-Environment Relations in the Out-of-Home Mobility of People Living With Dementia

**DOI:** 10.1093/geroni/igaa057.950

**Published:** 2020-12-16

**Authors:** Kishore Seetharaman, Habib Chaudhury, Atiya Mahmood

**Affiliations:** Simon Fraser University, Vancouver, British Columbia, Canada

## Abstract

This paper proposes a conceptual framework on the relationship between neighbourhood mobility and developmental outcomes of persons living with dementia. While there is growing evidence on the importance of out-of-home mobility for community-dwelling people with dementia, there is a lack of theoretical understanding of the person-environment (P-E) interactions involved in out-of-home mobility in the context of dementia and their influence on psychosocial outcomes. The proposed framework adapts Chaudhury and Oswald’s Integrative Conceptual Framework of Person-Environment Exchange to address the influence of out-of-home mobility-related P-E interactions on processes of agency and belonging for people living with dementia and the cumulative effect on developmental outcomes of autonomy and identity. The framework describes a linear sequence of four components: (i) out-of-home mobility-related P-E interactions determined by individual, social, physical environmental, and technological factors; (ii) mobility-related environmental barriers and facilitators and perception of out-of-home mobility-related risk and benefit; (iii) interrelated positive and negative processes of behaviour-driven agency and experience-driven belonging; and (iv) interrelated developmental outcomes of autonomy and identity. This framework advances our conceptual understanding of the relations between the experience of living with dementia and different aspects of the neighbourhood environment by bridging objective and subjective dimensions of P-E interactions and framing out-of-home mobility as an important contributor in maintaining identity and autonomy. The framework could inform empirical research in this area, evaluation of dementia-friendly community initiatives, and policy decisions by drawing attention to both the functional aspects and meanings associated with out-of-home mobility for persons living with dementia.

